# Catch-22: War, Refugees, COVID-19, and the Scourge of Antimicrobial Resistance

**DOI:** 10.3389/fmed.2022.921921

**Published:** 2022-06-24

**Authors:** Marwan Osman, Kevin J. Cummings, Khaled El Omari, Issmat I. Kassem

**Affiliations:** ^1^Cornell Atkinson Center for Sustainability, Cornell University, Ithaca, NY, United States; ^2^Department of Public and Ecosystem Health, College of Veterinary Medicine, Cornell University, Ithaca, NY, United States; ^3^Quality Control Center Laboratories at the Chamber of Commerce, Industry & Agriculture of Tripoli & North Lebanon, Tripoli, Lebanon; ^4^Laboratoire Microbiologie Santé et Environnement (LMSE), Doctoral School of Sciences and Technology, Faculty of Public Health, Lebanese University, Tripoli, Lebanon; ^5^Center for Food Safety, Department of Food Science and Technology, University of Georgia, Griffin, GA, United States

**Keywords:** antimicrobial resistance, COVID-19, refugees, civil war, armed conflict, displaced populations, infectious diseases, one health

## Abstract

Wars have hidden repercussions beyond the immediate losses of life, well-being, and prosperity. Those that flee wars and seek refuge in safer locations are not immune to the tragic impacts. Of particular concern is the susceptibility of the refugee populations to infectious diseases and antimicrobial-resistant pathogens. This poses a detrimental risk to these disenfranchised populations, who often have limited access to medical care, sanitation, and nutritious and safe food. Furthermore, antimicrobial-resistant pathogens in refugees can be both transmitted to and acquired from their hosting communities. The latter is particularly problematic when the host countries suffer from serious challenges such as limited resources, pollution, and widespread antimicrobial resistance (AMR). Here, we discuss AMR in refugees of the ongoing Syrian war, a conflict that resulted in the largest population displacement in recent history. We argue that Syrian refugees and their hosting communities are at an elevated risk of complicated and life-threatening AMR infections. We also call on the international community to address this grievous problem that threatens the disenfranchised refugee populations and can spill over across geographic borders to affect multiple countries.

## Introduction

Multiple conflicts are currently unfolding across the globe. However, the protracted Syrian civil war resulted in the largest refugee crisis in recent history. Due to political and economic unrest and armed conflicts, Syrian civilians have been seeking refuge in different parts of the world. Of notable concern is the proliferation of infectious diseases that ruthlessly exploit the unprecedented humanitarian conditions created by the Syrian conflict (1–5). For example, refugees are at high risk of susceptibility and/or exposure to life-threatening infectious diseases such as COVID-19 (6, 7), tuberculosis (8), hepatitis A (1), measles (3), and poliomyelitis (4). In many instances, the vulnerable and potentially immunocompromised refugees are exposed to conditions that favor the selection and acquisition of multidrug-resistant (MDR) infections, which also pose a heightened risk for a population with limited access to healthcare, immunizations, and essential medications (9–11).

Factors that favor the emergence of antimicrobial resistance (AMR) already existed in Syria before the conflict (11, 12). Although there are laws in Syria (number 2/T) that prohibit over-the-counter sale of antimicrobials and date back to 1988, Syria still experienced undeveloped and weakly enforced regulations and laws associated with the prudent use of antimicrobials (11). For example, concerns over losing clients were considered a primary driver that rendered antimicrobials readily accessible for purchase from pharmacies and agriculture drug stores without the need for a medical prescription (13). However, the ongoing Syrian conflict has created more favorable conditions for the selection and dissemination of AMR (11, 12). Indeed, existing reports on AMR in displaced Syrian populations have described increasing trends of MDR *Enterobacterales*, methicillin-resistant *Staphylococcus aureus* (MRSA), and drug-resistant tuberculosis (14–22). Taken together, it can be argued that there is an imminent threat to Syrian populations, especially refugees that live in camps with high exposure to pollution and limited access to water, sanitation, and hygiene (WASH) programs and adequate medical care. Additionally, the rise of AMR in Syrian populations has serious regional and global implications, because it can spill over to countries that host large populations of refugees.

A global health response is needed to implement comprehensive effective policies and intervention strategies to tackle the scourge of AMR in major locations and camps that host the refugees. However, this cannot be achieved without a deep understanding of the current epidemiology of AMR in these locations. Therefore, we aimed here to evaluate the threat of AMR in the context of the Syrian crisis. We also highlighted the humanitarian situation and AMR challenges that the Syrian refugees are facing inside Syria and in host countries, with a special focus on Lebanon, a country that is currently hosting ~ 1.5 million refugees while facing its own challenges with high drug resistance trends (23–26) and calamitous economic and political crises. These conditions have resulted in a shortage of medicine, appropriate diagnostic tools, water, and other critical necessities in Lebanon (27, 28).

## Antimicrobial Resistance in Brief: A Global Public Health Threat Facing Humanity

According to the World Health Organization (WHO), AMR is growing and has become one of the greatest public health challenges facing humanity (29, 30). In 2016, the Interagency Coordination Group on AMR predicted that drug-resistant infections could potentially cause up to 10 million deaths globally per year by 2050 (29). Perhaps predictably, the majority of the AMR-associated mortality and morbidity are projected to occur in countries with weak antimicrobial stewardship, low resources, and/or wide pollution. Furthermore, approximately five million deaths were associated with bacterial AMR in 2019; most of these cases included lower respiratory and bloodstream infections and a variety of etiologic agents such as *Escherichia coli, S. aureus, Klebsiella pneumoniae, Streptococcus pneumoniae, Acinetobacter baumannii*, and *Pseudomonas aeruginosa* that exhibited resistance to multiple classes of critical antibiotics (31). These studies at least shed some light on the bummock of the AMR iceberg.

The problem of AMR is further complicated by a faltering research and development pipeline with only a few new drugs on the horizon. The latter is due to many factors, which are mainly related to pharmacoeconomics and the challenges, financial and otherwise, associated with discovering and bringing new antimicrobials to the market (32). For example, the cost of developing an antibiotic can be around 33-fold higher than the yearly revenue generated from the respective drug sales (33). Consequently, humanity is facing increasingly recalcitrant infections without enough new drugs to control resistant pathogens. This raises many questions, including how will disenfranchised populations (e.g., refugees) with limited resources and access to proper medical care cope? What is the toll on the hosting country, especially if it is a low- and middle-income country (LMIC)? The judicious answer, at least partially, is to preemptively control AMR infections in these populations and their role as reservoirs for the emergence and spread of AMR pathogens. To achieve this, robust surveillance and monitoring studies are needed to understand the drivers of AMR in these populations. That is not an easy task given the magnitude of displacement, the disparate locations, conditions of refugee camps, and lack of resources, among a plethora of other issues.

## The Syrian Conflict and the Scope of Displacement

Millions of Syrians have fled violence and persecution to seek safety as displaced individuals both inside and outside Syria. According to the current estimations of the United Nations High Commissioner for Refugees, more than two-thirds of the Syrian population have been internally displaced in Syria (~ 6.7 million) and/or fled the country and sought asylum abroad (~ 6.6 million), noting that there are large numbers of unregistered refugees that are not listed in the census (https://www.unhcr.org/en-us/syria-emergency.html). Furthermore, the Syrian crisis has, directly and indirectly, affected several other countries in the Middle East and Europe. Notably, Turkey has been hosting the highest number of Syrian refugees (3.75 million), followed by Lebanon (~1.5 million), Jordan (0.67 million), Iraq (0.26 million), and Egypt (0.14 million). It is also estimated that one million Syrian asylum seekers and refugees have entered Europe, and the vast majority are hosted in Germany (59%) followed by Sweden (11%), while other European countries (Austria, Greece, the Netherlands, France, Kosovo, Serbia, Hungary, and Denmark) host between one to five percent.

Due to the massive influx of refugees, some European countries enforced laws and sanctions to prohibit refugees from seeking asylum on their lands. This along with the desperation to flee violence pushed the refugees to follow illegal and risky routes to enter Europe. The illicit entry caused a gap in the surveillance data and registry of Syrian families, further impeding them from accessing medical care, education, safe food, international funds, and humanitarian aid.

**Figure 1:**
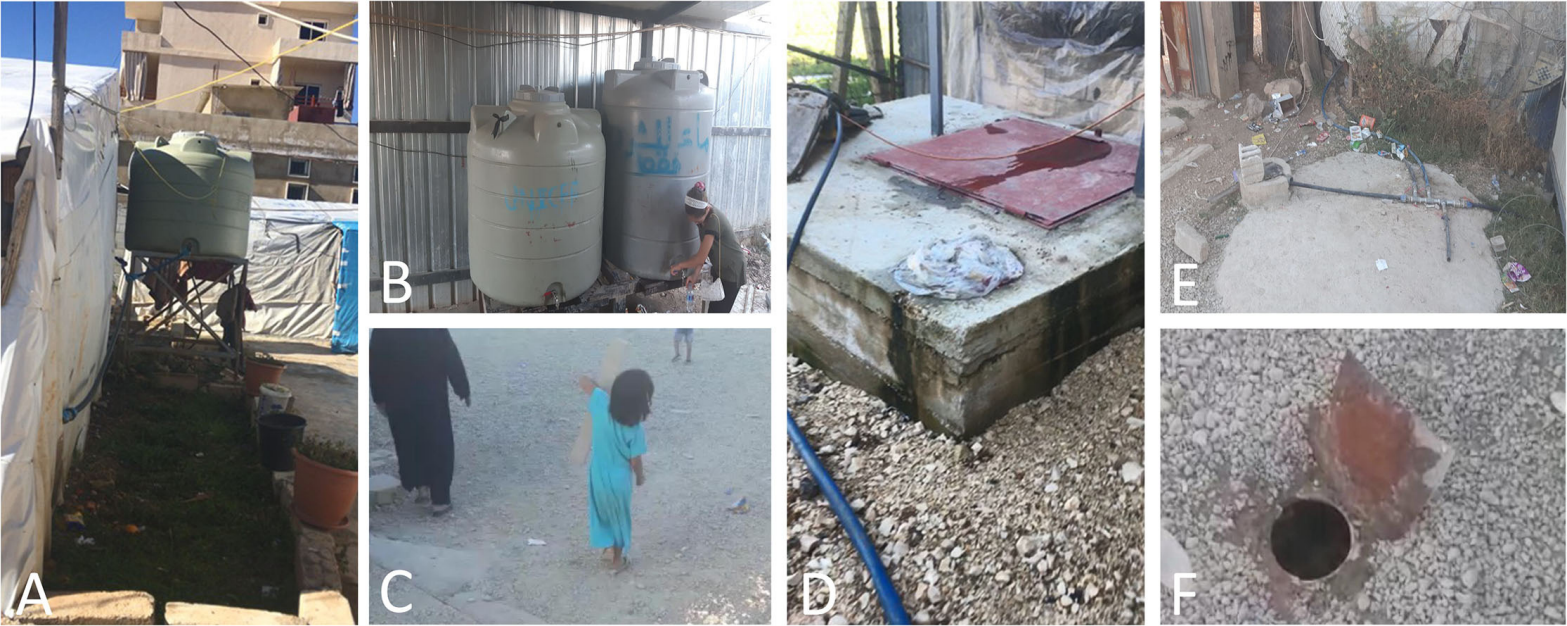
Photographic examples highlighting the conditions in refugee camps in Lebanon. White makeshift tents in proximity to hosting community residence (concrete building in the background) **(A)** and limited-capacity water cisterns **(A,B)** that provide domestic and drinking water to the camp inhabitants that include a large number of youths **(B,C)** and elderly individuals **(C)**. The cisterns are filled periodically via water trucks provided by non-governmental organizations (NGOs) and charitable donations. The source of the water varies, the supply is sporadic, and the cisterns are filled from underground cisterns/wells **(D,E)** when the trucked water supply is insufficient. The underground cisterns are very vulnerable (without proper protection) and prone to contamination from shallow and inappropriate camp sewage conduits **(F)**, especially after rainfall. The camp inhabitants have reported that the cisterns have been contaminated with sewage on multiple occasions. This spreads persistent contamination to drinking and domestic water cisterns. Camp environments are generally unclean (dust, stones, solid waste) as can be observed in all the pictures above. Photographs were documented by Mr. Abdallah AlHaj Sulaiman.

In many hosting countries, displaced Syrian populations live below the poverty line in makeshift tents and struggle to survive the elements and secure basic needs, depending largely on the support from the international community and the hosting countries. For instance, Syrian refugee households living in Lebanon are highly vulnerable and suffer from intense economic pressures (Figure 1). A large fraction of Syrian refugees continues to live in substandard residential (69%) and non-residential (11%) accommodations and informal tented settlements (20%) in Lebanon (34). In 2015, it was estimated that nearly 70% of Syrian refugee patients were forced to stop their medical treatments due to economic hardship (35). These deteriorating conditions have promoted the emergence of numerous public health problems, which overtaxed the fragile health systems in Lebanon. For example, the Lebanese National Tuberculosis Program (NTP) has shown a significant percentage of tuberculosis cases in Syrian refugees, leading to an increase in the incidences of tuberculosis in Lebanon (8). A nationwide study on the prevalence of tuberculosis in Lebanon described that approximately 30% of patients in the country were Syrians, and the Lebanese population only accounted for 28% of cases (22). Similarly, the Turkish Ministry of Health observed a noticeable increase in the percentage of imported tuberculosis cases, reaching approximately 7% in 2015 compared to 1.3% in 2011 (36). It is worth mentioning that the official data provided by the NTPs in Middle Eastern countries likely underestimate the actual rates of tuberculosis, particularly in areas experiencing economic and political collapse (11, 37). Taken together, these observations confirm that the prevailing conditions for large numbers of refugees are conducive for the acquisition of infectious diseases, especially in hosting countries that lack resources and face their own challenges.

## Antimicrobial Resistance Among Syrian Refugees: Evidence From Several Hosting Countries

Several reports have highlighted a high prevalence of drug-resistant etiologic agents among displaced Syrian populations and refugees and called for preemptive actions to tackle the problem. Notably, colistin, a last-resort antibiotic used to treat complicated infections, was effective on only 11% of the *P. aeruginosa* isolated from patients suffering from healthcare-associated infections in three major hospitals in Aleppo, Syria (38). Additionally, higher prevalence of MRSA and MDR *Enterobacterales* were found among Syrian refugees compared to the resident population in Germany, confirming previous findings from Finland, Denmark, Austria, Israel, Switzerland, and the Netherlands (11). Compared to German control populations, refugees from Syria showed large differences in the gut microbiota composition and an increased prevalence of AMR bacteria (39), where 6.3% and 1.6% of screened refugees carried MRSA and extended-spectrum cephalosporin-resistant *Enterobacterales* (ESCR-E), respectively (39). Another study in Germany found that patients admitted from refugee accommodations were notably more colonized with MRSA (10.3%) compared to German resident ICU patients without refugee history (1.4%) (40). Furthermore, 9.8% and 23.3% of the refugees exhibited colonization with MRSA and MDR Gram-negative bacteria in Frankfurt (Germany), respectively (41). MRSA is considered one of the top public health risks that affect minorities and immigrant populations and can cause invasive and life-threatening infections (42). Furthermore, resistance to extended spectrum cephalosporins (ESC) and carbapenems, which are also considered critically important antibiotics, is usually associated with resistance to other key antimicrobial agents. Losing the effectiveness of these antibiotic classes usually leads to more labor-intensive treatments and, in some cases, futile interventions (43).

It is important to note that the predominant proportion of new sensitive and resistant tuberculosis cases across the world are among immigrants, asylum seekers, and refugees (44). These populations have a heightened susceptibility to drug-resistant tuberculosis associated with worse clinical outcomes (21). For example, the first description of an extensively drug-resistant (XDR)-tuberculosis case in Lebanon was among refugees or asylum seekers from Syria, Sudan, and Ukraine (22). Although limited data have been published on the emergence and spread of MDR and XDR tuberculosis among Syrian refugees in host countries, drug-resistant isolates from Syrian refugees were reported in Lebanon (22), Jordan (45), Turkey (46), Germany (47), and the Netherlands (48, 49). Obviously, the transmission of MDR tuberculosis in these populations and during refugee migration (spread to hosting countries) poses a high public health priority for all stakeholders (50).

The spread of AMR in refugee populations is also evident in some of their ill-constructed camps and pollution-susceptible necessities such as domestic water. Studies have shown that the camp environment can be polluted and lead to an increased exposure of refugees to AMR pathogens and determinants (51, 52). The first data on the occurrence of colistin resistance and the mobile colistin resistance gene (*mcr-1*) in Syrian refugee camps were described in Lebanon, highlighting the detection of OXA-48 and KPC carbapenemases in some colistin-resistant isolates (53). Notably, the *mcr* gene was later found to be plasmid-borne, suggesting that it can transmit colistin resistance between bacterial species (54). Furthermore, genomic analysis of two of the *mcr* containing isolates showed the presence of an additional 14 to 19 AMR genes that encoded resistance to several classes of antibiotics, including aminoglycosides, diaminopyrimidines, macrolides, β-lactams, phenicols, fosfomycin, tetracyclines, fluoroquinolones, and sulfonamides (54). Colistin-resistant *E. coli* isolates from sewage and domestic water collected from the camps exhibited co-resistance to multiple other antimicrobials, including ESC (22–64%), carbapenems (6%), tetracycline (94%), chloramphenicol (94%), fluoroquinolones (75–86%), and trimethoprim-sulfamethoxazole (92%). In another study, molecular analysis of MDR *E. coli* isolated from sewage water in Syrian refugee camps located in the Beqaa Valley revealed that 53.1% of isolates were positive for numerous resistance determinants (such as CTX-M-14, OXA-1, SHV-12, CMY-2, aac(6)-Ib, acc(3)-II, and Int-I1) (55). The infrastructure in refugee camps is non-existent or weak at best, and makeshift sewage conduits in the camps were shallow and exposed. During heavy rains, these sewage outlets overflooded and leaked into domestic water cisterns, eventually contaminating drinking water. These studies were followed by investigations that reported for the first time the detection of *mcr-1* in *Proteus mirabilis* isolated from domestic and sewer water in Syrian refugee camps in Lebanon (56). *P. mirabilis* is an opportunistic pathogen that can cause a variety of illnesses, including complicated urinary tract infections that can lead to bacteremia. Taken together, these observations suggested that important AMR determinants may be circulating in different bacteria in the camp environment, and perhaps also being amplified in the refugees. The latter indicates a cycle of amplification and transmission between refugees and their camp environments. Notably, the untreated sewage generated from the camps in Lebanon ends up in nearby rivers or affects agricultural water channels (irrigation), posing a risk of transmission to the hosting community (57).

It should be noted that refugees are also exposed to AMR determinants circulating in the hosting country. This can be significant when considering the AMR trends in these countries. For example, in Lebanon, a recent report highlighted that the prevalence of *mcr-1*-positive MDR *E. coli* detected in Lebanese irrigation water was high (58). Also, the occurrence and persistence of transmissible *mcr-1* in colistin-resistant *E. coli* has been reported for the first time in the Mediterranean Sea along the coast of Lebanon (59). Additionally, another report showed that Lebanese rivers suffer from elevated fecal pollution and *E. coli* isolated from these waters exhibited resistance to ampicillin (40% of isolates), amoxicillin-clavulanic acid (42%), cefepime (4%), cefotaxime (14%), cefalexin (46%), cefixime (17%), doripenem (0.3%), imipenem (0.5%), gentamicin (6%), kanamycin (9%), streptomycin (35%), tetracycline (35%), ciprofloxacin (10%), norfloxacin (7%), trimethoprim-sulfamethoxazole (32%), and chloramphenicol (13%) (60). Of particular concern was that 45.8% of the isolates were MDR. These observations are significant, because many refugee camps use river water for domestic purposes, especially when water supplies (via donations or non-governmental organizations) to the camps are limited or unavailable. Furthermore, many camps are located in agricultural areas in close proximity to irrigation water, and many refugees seek recreation on freely-accessible public beaches and rivers in Lebanon.

**Figure 2:**
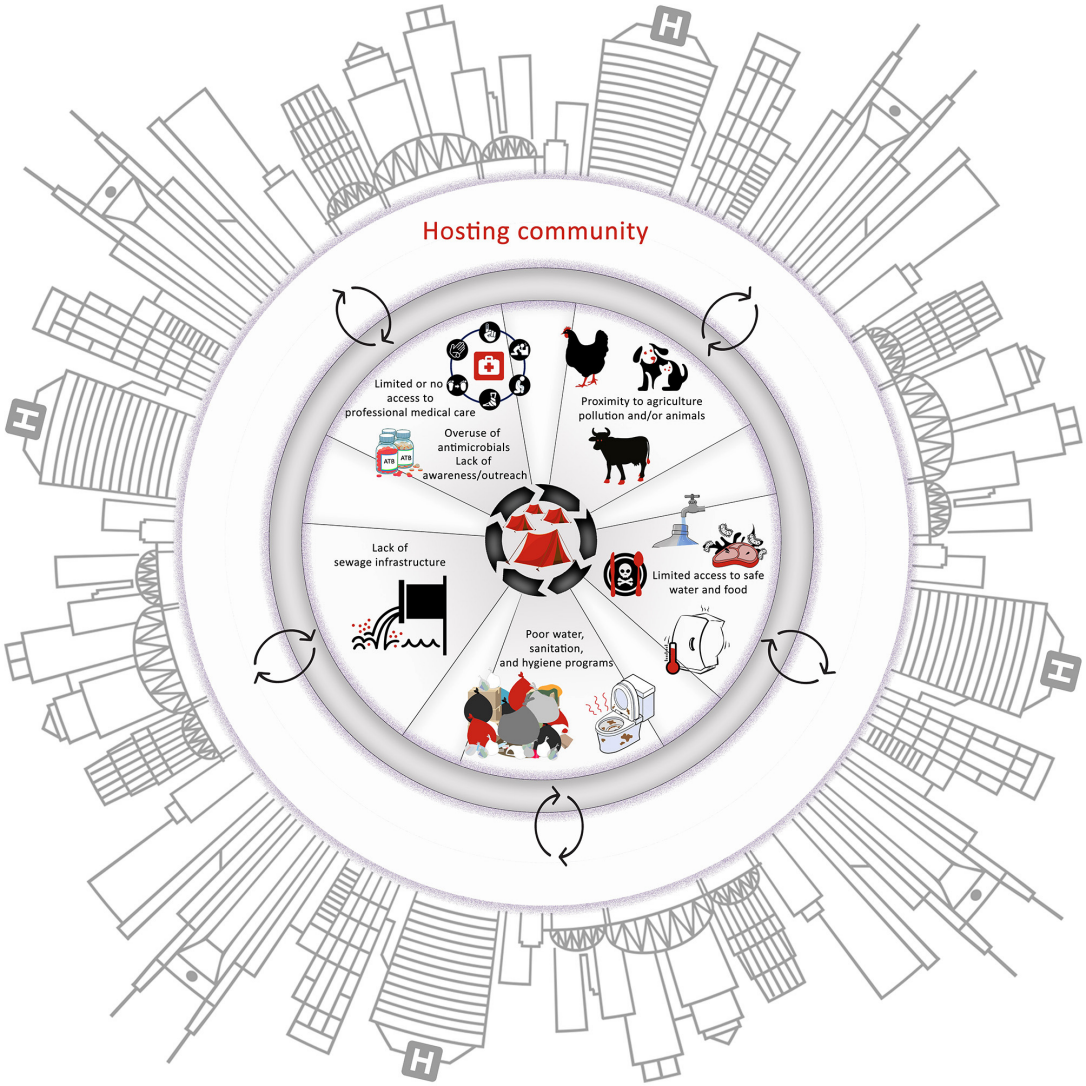
The major factors that influence the emergence and spread of antimicrobial-resistant pathogens in the camps. It is important to note the interactions between all the factors and between the camps and the hosting community. These interactions can create suitable conditions for the emergence and rapid spread of antimicrobial resistance (AMR) within camps and hosting communities, highlighting the necessity to adopt a One Health approach to tackle AMR in these scenarios.

Taken together, the susceptibility of refugee populations to AMR infections and their role in transmission of resistant pathogens across borders should not be viewed independently from the AMR trends in the hosting country. Again, the latter is especially true in LMICs that have widespread pollution and proliferation of AMR determinants. Stakeholders should be cognizant of the transmission dynamics of AMR in both refugees and the hosting countries (Figure 2); otherwise, interventions to control AMR in these populations might have a limited impact.

## COVID-19, a Failing Economy, and the Emergence of Infectious Diseases and AMR in Syrian Refugees: A Focus on Lebanon

The healthcare system in Lebanon poses multiple challenges for refugees, including a lack of clarity, mistrust, discrimination, and limited and fragmented services. Although health services are in principle available for registered and unregistered Syrian refugees in Lebanon for an affordable fee (61), 13% of patients had no access to any primary healthcare services and only 81% of patients seeking hospital care were able to receive the needed services (11). Approximately, two-thirds of the refugees have been reported to stop the use of medications due to the inability to afford medicines or handling fees (35). Notably, these challenges existed prior to the onset of COVID-19 and the collapse of the Lebanese economy.

Since October 2019, Lebanon has been witnessing an unprecedented economic collapse and inflation, which were further exacerbated by the emergence of the COVID-19 pandemic. Lebanon has experienced a large epidemic expansion (as of April 13, 2022, there were 1,094,911 cases as well as 10,350 COVID-19 deaths). Furthermore, the devaluation (> 90%) of the Lebanese pound has increased medical costs and pushed more than half of the resident populations, Lebanese and refugees, into extreme poverty. These populations have become unable to purchase personal protective equipment (e.g., masks, disinfecting products) or afford food and health services. Notably, Lebanon also relies heavily on imports to meet most of its food, energy, and medical needs (27). The inability to cover or even subsidize essential medications became an additional barrier to seeking healthcare for these vulnerable populations.

Given that the Lebanese government has immensely struggled to cope with the pandemic and provide care for Lebanese citizens, refugees were by default at heightened risk for SARS-CoV-2 infections. Weak public health interventions have been reported in refugee camps since the onset of the Lebanese economic crisis (62). United Nations data revealed that Syrian refugees have died from COVID-19 at a rate more than four times the national average (63). The living conditions of Syrian refugees in overcrowded shelters with scarce access to WASH and infection prevention and control (IPC) services likely allowed the occurrence of greater and more severe infectious disease outbreaks in high-density refugee camps, including SARS-CoV-2 outbreaks (57). Subsequently, to cope with challenges related to the lack of oxygen supply and limited healthcare, more antimicrobials were inappropriately used to treat COVID-19 patients (refugees and Lebanese patients). Indeed, anecdotal evidence suggested an overreliance on self-medication with azithromycin and other drugs in these populations. To date, there are no data on the scale of antimicrobial use in these settings during the pandemic. However, healthcare professionals have voiced great concerns about the inappropriate use of antimicrobials and unjustified prolonged antimicrobial treatments of patients infected with COVID-19 in Lebanon (64–67). Therefore, an increase in the burden of AMR has been predicted in Syrian refugees and other vulnerable populations in Lebanon as a result of both COVID-19 and the dire social and economic situation.

## Conclusions

Displacement, dire living conditions, infectious disease, and AMR are real challenges that the Syrian refugees face on a daily basis. To prevent the exacerbation of these challenges and avoid catastrophic consequences on the refugees and their vulnerable hosting communities, engagement of stakeholders across the globe and investments in infrastructure and One Health approaches are critical. AMR surveillance systems and antimicrobial stewardship programs using genomic and machine-learning analyses are also needed to mitigate the sources of AMR affecting refugees and hosting communities and to optimize their access to necessary antimicrobials. International aid organizations must be cognizant of the transmission dynamics of AMR and play a key role in helping to implement reliable action plans to control AMR in both the refugees and hosting countries. The latter is critical in countries like Lebanon that suffer from limited resources and a variety of healthcare and economic challenges. It should be noted that the challenges discussed in this manuscript are not limited to Syrian refugees and can be experienced by other refugees such as the Rohingya, South Sudanese, and Congolese among others.

## Data Availability Statement

The original contributions presented in the study are included in the article/supplementary material, further inquiries can be directed to the corresponding authors.

## Author Contributions

MO and IIK contributed to the conception of the study and data acquisition and drafted the manuscript. IIK supervised the work. KC and KE critically revised the manuscript. All authors contributed to the article and approved the submitted version.

## Funding

MO is supported by the Atkinson Postdoctoral Fellowship (Cornell University). The work was partially supported by funds from the Center for Food Safety (University of Georgia).

## Conflict of Interest

The authors declare that the research was conducted in the absence of any commercial or financial relationships that could be construed as a potential conflict of interest.

## Publisher's Note

All claims expressed in this article are solely those of the authors and do not necessarily represent those of their affiliated organizations, or those of the publisher, the editors and the reviewers. Any product that may be evaluated in this article, or claim that may be made by its manufacturer, is not guaranteed or endorsed by the publisher.
